# Tumour promoting and suppressing roles of the atypical MAP kinase signalling pathway ERK3/4-MK5

**DOI:** 10.1186/1750-2187-7-9

**Published:** 2012-07-16

**Authors:** Sergiy Kostenko, Gianina Dumitriu, Ugo Moens

**Affiliations:** 1 Department of Medical Biology, Faculty of Health Sciences, University of Tromsø, Tromsø, NO-9037, Norway

**Keywords:** PRAK, RAS, c-MYC, IGB2PB, Angiogenesis, Senescence, HSP27, FOXO3a

## Abstract

Perturbed action of signal transduction pathways, including the mitogen-activated protein (MAP) kinase pathways, is one of the hallmarks of many cancers. While the implication of the typical MAP kinase pathways ERK1/2-MEK1/2, p38^MAPK^ and JNK is well established, recent findings illustrate that the atypical MAP kinase ERK3/4-MK5 may also be involved in tumorigenic processes. Remarkably, the ERK3/4-MK5 pathway seems to possess anti-oncogenic as well as pro-oncogenic properties in cell culture and aninal models. This review summarizes the mutations in the genes encoding ERK3, ERK4 and MK5 that have been detected in different cancers, reports aberrant expression levels of these proteins in human tumours, and discusses the mechanisms by which this pathway can induce senescence, stimulate angiogenesis and invasiveness.

## Introduction

### Mitogen-activated protein kinase pathways

**Figure 1:**
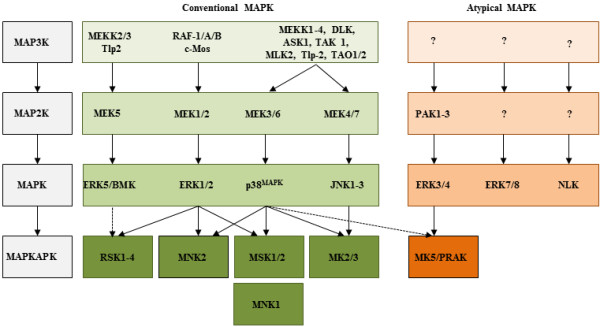
**Schematic presentation of the different mammalian MAP kinase pathways.** The conventional MAP kinase pathways, represented by the MEK/ERK, JNK, p38^MAPK^, and MEK5/ERK5 pathways, consist of a module of three kinases that subsequently phosphorylate and active each other. The MAPK kinase kinase (MAP3K) phosphorylates MAPK kinase (MAP2K), which in turn phosphorylates MAPK. Downstream of MAPK are substrates including other protein kinases referred to as MAPK-activated protein kinases (MAPKAPK). The atypical pathways include ERK3, ERK4, ERK7, ERK8, and NLK. MAPKs can converge to different MAPKAPK as shown in this figure.

The mitogen-activated protein kinase (MAPK) pathways play crucial roles in cell proliferation, differentiation, gene expression, apoptosis, metabolism and motility [1-5]. A typical MAPK pathway consists of a cascade of three consecutive phosphorylation events exerted by serine/threonine kinases known as MAPK kinase kinase (MAPKKK or MAP3K), a MAPK kinase (MAPKK or MAP2K), and a MAPK (Figure 1, [1-5]).

Substrates of MAPKs include protein kinases designated as MAPK-activated protein kinases (MAPKAPK). The human MAPKAPK family comprises the ribosomal-S6-kinases (RSK1-4), the MAPK-interacting kinases (MNK1 and 2), the mitogen-and stress-activated kinases (MSK1 and 2), and MAPKAPKs MAPKAPK-2 (MK2), MAPKAPK-3 (MK3), and MAPKAPK-5 (MK5) [6-10]. RSKs are downstream targets of the extracellular signal-regulated kinases ERK1, ERK2 and ERK5, and regulate cell growth, cell proliferation, cell survival, transcription and translation, and cell motility [5,11-13]. MNK1 and MNK2, which are activated *in vivo* by ERK1/2 and p38^MAPK^, participate in transcriptional and translational regulation, inflammatory responses, proliferation and survival [8,14]. MSK-1 and MSK-2 operate downstream of ERK1/2 and p38^MAPK^, and play versatile roles in transcription, translation, inflammatory responses, and neuronal processes [7,10]. Of MK2, MK3 and MK5, MK2 and MK3 are most closely structurally and functionally related. They share 75% overall sequence identity and are directly activated by p38^MAPK^[6,15]. MK2 participates in cytokines production, endocytosis, cytoskeleton architecture, cell migration, cell cycle control, cell survival, and transcriptional regulation and may play a causal role in infections and inflammatory diseases such as rheumatoid arthritis, atherosclerosis, and asthma [6,14-16]. The precise role of MK3 is not fully understood because MK3 deficient mice are viable and display no obvious phenotype. However, studies in a MK2-deficient background indicated that MK3 can compensate for the loss of MK2, while MK3 seems to inhibit interferon-γ expression by impairing interferon regulatory factor 3 protein expression and activation and inhibiting nuclear translocation of the p65 subunit of NFκB [17,18].

### MAPK ERK3 and ERK4

The human ERK3 (~100 kD) and ERK4 (~70 kD) proteins have a similar structural organization and are 73% identical in their kinase domain. Both are atypical MAPK because their activation loop lacks a phosphoacceptor Tyr residue (conserved TXY motif), but contains a Ser-Glu-Gly (SEG) motif [5,19]. ERK4 is a relative stable protein, while ERK3 is rapidly degraded via the ubiquitin-proteasome pathway [20-23]. When overexpressed in cells, both ERK3 and ERK4 redirect MK5 exclusively to the cytoplasm [20,21,24]. ERK3 and ERK4 can be activated by the group I p21-activated protein kinases PAK1, PAK 2, and PAK3 [25,26].

ERK3 deficient mice on a C57BL/6 background are non-viable [27]. Approximately 40% of the born offspring die within 15 min of delivery because of acute respiratory failure, while the survivors display severe phenotypic changes such as uncoordinated movements, lack of reflex on pinching, infirm vocalization, and diminished suckling reflex. The surviving animals die within 24 h after birth. ERK3^−/−^ mice have foetal growth restrictions and reduced body and organ weight, but no gross morphological changes. Moreover, they are characterized by pulmonary hypoplasia and incomplete differentiation of type II pneumocytes, confirming a role of ERK3 in differentiation and proliferation. ERK3^−/−^ mice display also defects in production and/or secretion of insulin-like growth factor II [27].

Disruption of the *mapk4* gene encoding ERK4 has no obvious effects on viability, fertility, morphology and physiology of the mice on a mixed C57BL/6 J × 129/Sv genetic background [28]. Moreover, knockout of the *mapk4* gene in ERK3 deficient mice does not exacerbate their phenotype. However, ERK4-deficient animals on a C57BL/6 J genetic background possess increased depression-like behaviour [28]. The cellular and physiological roles of ERK4 remain obscure.

### Mitogen-activated protein kinase-activated protein kinase-5 (MK5)

MK5 is a 54 kD ser/thr kinase that can be activated *in vitro* by ERK2, JNK and p38^MAPK^ through phosphorylation of Thr-182 [29,30]. MK5 is ubiquitously expressed, but is most abundant in brain, heart and platelets [29-31]. The organization of the gene and primary sequence of the protein are well conserved in vertebrates, but a gene encoding MK5 seems absent in invertebrates [32]. MK5 has a functional nuclear localization signal and nuclear export signal that allows the protein to shuttle between the nucleus and the cytoplasm. In resting cells, MK5 is predominantly found in the nucleus [24,33]. The N-terminal region of MK5 contains the catalytic domain, while the C-terminal region is required for ERK3 and ERK4 binding. MK5 also contains a p38^MAPK^ docking motif, which overlaps with the nuclear localization signal [32].

Although the amino acid sequence and the structural organization of MK5 show most similarity to MK2 and MK3 (approximately 33% homology,), several unique properties clearly distinguish MK5 from MK2/MK3. MK5 has a unique C-terminal sequence which is lacking in MK2 and MK3 [5,6,30,32]. In contrast to MK2 and MK3, MK5 belongs to the atypical MAPK pathways (Figure 1, [5]). ERK3 and ERK4 are upstream activators of MK5 that can induce phosphorylation of MK5 at Thr-182 [20,21,34-36]. PAK-induced activation of ERK3/4 results in increased phosphorylation of MK5 at Thr-182 and stimulated the enzymatic activity of MK5 [25,26]. There is controversy to whether p38^MAPK^ can phosphorylate MK5 on Thr-182 *in vivo*, but recent results show that the p38β isoform can activate MK5 *in vivo*[37,38]. Another activator of MK5 is cAMP-dependent protein kinase (PKA) which phosphorylates MK5 at Ser-115 [24,39].

The biological role of MK5 remains incompletely understood. MK5 knockout mice generated on a mixed 129 × C57/B6 genetic background are viable, fertile and display no obvious phenotype, while *mk5*^−/−^ mice on a C57BL/6 genetic background show embryonic lethality with incomplete penetrance around E11 [35,40]. Transgenic mice overexpressing a constitutive active mutant of MK5 display sex-specific changes in anxiety behaviour and locomotor activity, but the underlying molecular mechanisms have not been identified [41].

## Association of ERK3/4-MK5 pathway with cancer

### Effect of ERK3, ERK4 and MK5 on cell proliferation

**Figure 2:**
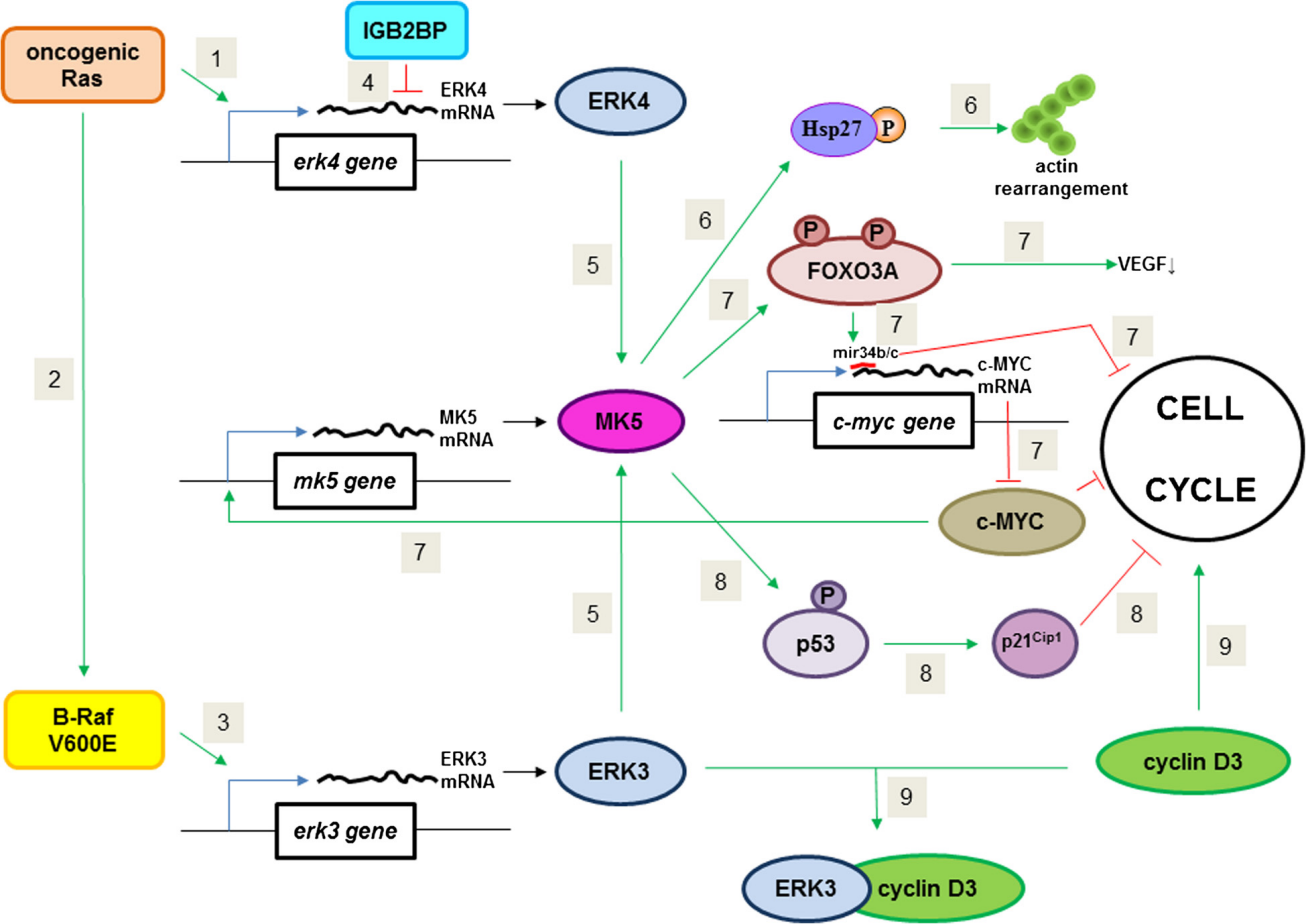
**Molecular mechanisms of the pro- and anti-oncogenic properties of ERK3, ERK4, and MK5.** (1,2) Oncogenic Ras mutants can increase the transcript levels of ERK4, but also results in constitutive activation of Raf. (3) Activated Raf or oncogenic BRAFV600E also stimulates transcription of the *erk3* gene. (4) The RNA binding protein IGB2BP can inhibit translation of ERK4 mRNA. (5) ERK3 and ERK4 proteins can activate MK5. (6) Active MK5 can phosphorylate Hsp27, which will affect actin remodelling and cell migration. (7) MK5 can also phosphorylate transcription factor FOXO3a, which in turn will trigger transcription of microRNA mir34b/c. mir34b/c inhibits the cell cycle and prevents translation of c-*myc* mRNA and hence the production of c-MYC, a protein that is important for cell cycle regulation. C-MYC binds to the promoter of the *mk5* gene and enhances transcription of this gene. FOXO3a also reduces expression of VEGF and may thus hamper angiogenesis. (8) MK5-mediated phosphorylation of p53 at Ser-37 stimulates the transcriptional activity of p53, resulting in enhanced expression of p21^Cip1^. p21^Cip1^ inhibits cell cycle progression. (9) ERK3 can sequester cyclin D3. By usurping cyclin D3, it may cause cell cycle arrest.

**Figure 3:**
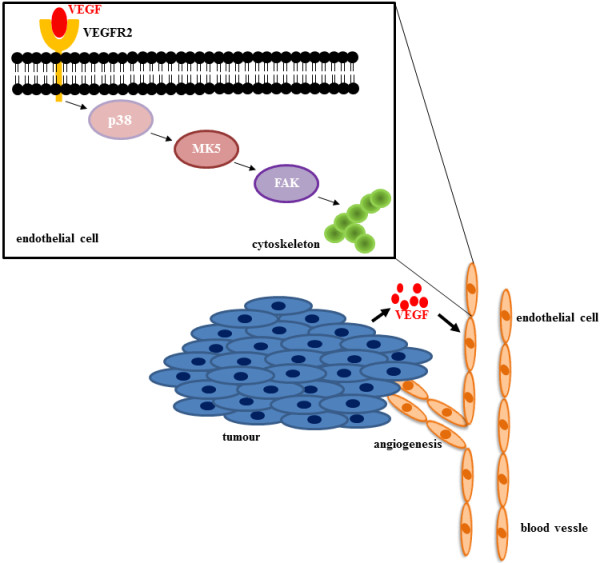
**MK5 is implicated in VEGF-mediated angiogenesis. VEGF produced by tumour cells will bind to the VEGF receptor VEGFR2 on endothelial cells.** This will trigger a cascade involving the MAP kinases p38 and MK5. MK5 can in turn activate FAK, and this will result in changes in the cytoskeletal architecture and migration of endothelial cells towards the tumour. A blood vessel with endothelial cells aligning the vessel is shown. The pathway induced by VEGF and which involves MK5 is depicted in detail.

Several observations disclose an involvement of the ERK3/4-MK5 pathway in cell cycle regulation (Figures 2 and 3). Elevated ERK3 levels result in G1 cell cycle arrest and inhibition of cell proliferation [22,42,43]. So far, it is not known whether ERK4 is implicated in cell cycle regulation because no difference in the proliferation rate between wild-type and ERK4^−/−^ fibroblasts was observed [28]. Overexpression of wild-type, but not kinase dead MK5 inhibited oncogenic H-Ras induced cell proliferation of NIH3T3 cells [44]. Ectopic expression of MK5 alone also reduced proliferation of NIH3T3 cells [45]. Oncogenic H-Ras-induced senescence is compromised in MK5 deficient primary murine fibroblasts and embryonic fibroblasts. Wild-type MK5, but not a kinase-dead mutant restored the senescence response to oncogenic Ras, indicating that the enzymatic activity of MK5 is required [46]. Knockout of the *mk5* gene in mouse embryonal fibroblasts leads to cell cycle arrest and proliferation inhibition [47].

### Somatic mutations in the erk3, erk4 and mk5 genes in human tumour tissues

A limited number of samples of a restricted selection of cancers have been examined for mutations in the *erk3, erk4* and the *mk5* gene. Silent mutations or single amino acid substitutions have been detected in lung, ovary and skin cancer tissue for ERK3, in lung and skin cancer tissue for ERK4, and in lung, melanoma and ovary cancer samples for MK5 (Tables 1,2,3; [48-54]). The effect of these mutations on the biological properties of ERK3, ERK4 and MK5 needs to be investigated, and it is has to be determined whether these are driver or passenger mutations. Interestingly, the L290V substitution in ERK3 was observed in both lung and skin cancer samples [48,51]. The E331D and D336N substitutions in ERK4 are located in the MK5 interaction motif [55]. In an overlay assay using ERK4 peptides with wild-type sequence or single amino-acid substitutions bound to cellulose membrane, the authors showed that D336N, but not E331D reduced *in vitro* binding of recombinant MK5. This observation may indicate that the *in vivo* interaction between ERK4 D336N and MK5 is weakened and affects the ERK4-MK5 signalling. ERK4 mutations A52V and P246T reside in the kinase domain, but it is not known if they affect the catalytic activity of ERK4.

**Table 1:** Somatic mutations in mapk6/erk3 in cancer tissues

**Primary tissue**	**Unique mutated samples**	**% mutated**	**Total unique samples**	**Mutation data**	**references**
bilary tract	0	0%	2		
breast	0	0%	201		
CNS	0	0%	477		
haematopoietic and lymphoid tissue	0	0%	27		
kidney	0	0%	28		
large intestine	0	0%	42		
lung	3	1%	359	c.190 C > T : R64C	[48]
				c.700 G > T: G234C	[48]
				c.868 C > G: L290V	[48]
ovary	1	1%	85	c.1466A > C: K489T	[50]
pancreas	0	0%	31		
pleura	0	0%	1		
prostate	0	0%	58		
salivary gland	0	0%	9		
skin	2	25%	8	c.868 C > G: L290V	[51]
				c.1522 G > A: E508K	[51]
stomach	0	0%	34		
testis	0	0%	13		
upper aerodigestive tract	0	0%	19		
urinary tract	0	0%	2		

**Table 2:** Somatic mutations in mapk4/erk4 in cancer tissues

**Primary tissue**	**Unique mutated samples**	**% mutated**	**Total unique samples**	**Mutation data**	**references**
bilary tract	0	0%	2		
breast	0	0%	201		
CNS	0	0%	477		
haematopoietic and lymphoid tissue	0	0%	27		
kidney	0	0%	28		
large intestine	0	0%	42		
lung	4	1%	359	c.354 G > A : Q118Q	[49]
				c.736 C > A: P246T	[48]
				c.993 G > T: E331D	[49]
				c1006G > A: D336N	[48]
ovary	0	0%	84		
pancreas	0	0%	31		
pleura	0	0%	1		
prostate	0	0%	58		
salivary gland	0	0%	9		
skin	3	33%	9	c.155 C > T: A52V	[53]
				c.340 C > T: R114C	[51]
				c.812A > G: K271R	[53]
stomach	0	0%	34		
testis	0	0%	13		
upper aerodigestive tract	1	5%	20	c.841 G > A: V281M	[52]
urinary tract	0	0%	2		

**Table 3:** Somatic mutations in mapkapk-5 in cancer tissues

**Primary tissue**	**Unique mutated samples**	**% mutated**	**Total unique samples**	**Mutation data**	**references**
bilary tract	0	0%	2		
breast	0	0%	201		
CNS	0	0%	477		
haematopoietic and lymphoid tissue	0	0%	27		
kidney	0	0%	28		
large intestine	0	0%	42		
lung	2	1%	171	c.406 C > T: L136L	[49]
				c.450 G > A: K150K	[49]
				c.1371A > G: E457E	[49]
melanoma	1	4%	25	c.890 G > A: L297E	[54]
ovary	1	1%	85	c.660 + 2 T > A: intronic mutation	[50]
pancreas	0	0%	31		
pleura	0	0%	1		
prostate	0	0%	58		
salivary gland	0	0%	9		
skin	0	0%	6		
stomach	0	0%	34		
testis	0	0%	13		
upper aerodigestive tract	0	0%	19		
urinary tract	0	0%	2		

### Altered expression of ERK3, ERK4 or MK5 in cancer tissue

The expression of proliferating cell nuclear antigen (PCNA), a key regulator in cell cycle control, DNA replication and repair, is strongly upregulated in many chronic myelogenous leukaemia (CML) patients. siRNA-mediated depletion of PCNA expression in several CML cell lines resulted in down-regulation of PAK2 and ERK3 transcript levels [56]. This may suggest that in CML patients with enhanced PCNA expression, the levels of PAK2 and ERK3 are also elevated and these perturbed expression levels may be implicated in the development of CML. Rai and colleagues examined the levels of ERK3 transcripts in oral tissue and peripheral blood cells from patients with chewing-tobacco-induced oral squamous cell carcinoma and healthy controls [57]. They found that 37/41 (90%) of the patients examined displayed a 5- to 8-fold increase in ERK3 transcripts in oral cancer tissue compared to healthy tissue. Peripheral blood cells of 8/13 (61.5%) oral squamous cell carcinoma patients also showed increased ERK3 mRNA levels, compared to absence or low levels in 71% (23/31) in peripheral blood cells of healthy individuals. Sequencing of the *erk3* gene of oral squamous cell carcinoma tissue revealed that the coding sequence was identical with the sequence of the *erk3* gene determined from foetal skeletal muscle. However, the oral cancer *erk3* gene sequence had 4 nucleotide alterations in the non-coding region. Although it was not tested whether all oral cancer patients with increased ERK3 transcript levels carried the same mutations in their *erk3* gene, the authors speculated that these mutations may affect the stability or translation efficiency of ERK3 mRNA, resulting in overexpression of ERK3. The presence of relatively high ERK3 transcripts in peripheral blood of healthy individuals may indicate that these individuals are at higher risk of developing oral cancer [57].

Oncogenic mutations in the *b-raf* gene, especially activating V600E mutation, is very common in several forms of cancer [58,59]. Inducible expression of oncogenic BRaf^V600E^ in NIH3T3 cells resulted in ~10-fold increased transcript levels of ERK3 and augmented protein levels compared to control cells [60]. Studies with proteasomal inhibitors demonstrated that BRaf^V600E^ exclusively regulates ERK3 expression at the transcriptional level without affecting proteasome-mediated turnover of ERK3 protein. The mechanism by which BRaf^V600E^ enhances ERK3 transcription is not known, but knockdown of BRaf^V600E^ in the melanoma cell line A375 or inhibition of MEK1/2, but not p38^MAPK^ by respectively U0126 and SB203580, abrogated ERK3 expression. Whether ERK3 contributes to tumorigenesis in melanoma expressing BRaf^V600E^ remains elusive because depletion of ERK3 in A375 cells did not change tumour cell proliferation, apoptosis or angiogenesis in mice injected with A375 cells stably transfected with ERK3 shRNA [60]. PAK3 is an upstream activator of ERK3 [25,26]. Pak3 mRNA levels, however, were downregulated upon induction of BRaf^V600E^ expression [60]. These results indicate that BRaf^V600E^-induced transcriptional upregulation of the *erk3* gene is independent of PAK3. Wang and co-workers found that 10/21 colorectal cancer tissues had higher ERK3 expression levels that adjacent normal mucosa [61]. Elevated ERK3 protein levels are also associated with gastric cancer. The protein levels of ERK3 were on average 3.85-fold higher in 27/42 gastric cancer tissues compared with paired adjacent normal mucosa samples [62]. ERK3 levels were higher in stage II and stage III tumours than in stage I and stage IV tumours. Metastatic lymph nodes (n = 14) had on average 6.9-fold higher ERK3 protein levels than adjacent mucosa specimens [61]. Determination of relative ERK3 transcript levels by quantitative real-time PCR in 48 mouse salivary gland tissues (7 normal, 13 dysplasias, and 28 adenocarcinomas) showed increased levels in dysplasia and adenocarcinomas compared to normal tissue [63]. Increased ERK3 transcripts or protein levels have also been observed in breast cancer, melanoma and non-small cancer lung cells [64-66]. It remains to be established whether increased ERK3 levels contribute to oncogenesis.

Less is known about ERK4 and MK5 protein levels in cancers. Lung adenomas in oncogenic K-Ras transgenic mice contain increased ERK4 levels compared with lungs of non-transgenic mice, but the biological relevance is not known [67]. To our best knowledge, no aberrant MK5 protein levels in tumours have been reported so far.

## Molecular mechanisms of the oncogenic potentials of the ERK3/4-MK5 signalling pathway

The fine molecular mechanisms by which ERK3 prevents cell cycle progression are incompletely understood, but ERK3 binds cyclin D3, as well as Cdc14A (an antagonist of cyclin-dependent kinase 1), while Cdc14A stabilizes complex formation between ERK3 and cyclin D3 [68]. ERK3 may sequester cyclin D3, thereby inhibiting cell cycle progression [68].

Long et al. [66] elegantly demonstrated that ERK3 promotes lung cancer cell invasion *in vitro* and *in vivo* through upregulating the expression of matrix metalloproteinase MMP2 and MMP10. ERK3 enhances transcription of the *MMP2* and *MMP10* genes by phosphorylating the steroid co-activator SRC-3 at Ser-857, which is essential for the interaction of SRC-3 with transcription factor PEA3. *MMP2* and *MMP10* are SRC-3/PEA3 target genes [66].

A causal role for ERK4-MK5 in metastasis was recently proposed [69]. Insulin-like growth factor 2 mRNA-binding protein (IGF2BP, also known as IMP, VICKz or ZBP1) belongs to a family of highly conserved proteins that regulate stability, location, and translation of a subset of mRNAs and as such, participate in cell polarity and migration [70,71]. IMPs are overexpressed in various human cancers where they are implicated in the formation of lamellipodia and invadopodia, and thus may contribute to metastasis [69,72-74]. A recent study by Stöhr and co-workers unveiled a role of the ERK4-MK5 pathway in IGF2BP- induced tumour cell migration [69]. Microarray analysis of total RNA isolated from stressed osteosarcoma U2OS cells upon IGF2BP1knockdown identified 74 annotated transcripts decreased ≥8-fold compared to control cells. One of the transcripts encodes ERK4. The authors showed that IGF2BP1prevents translation of ERK4 mRNA, which antagonizes MK5 activation and HSP27 phosphorylation. Knockdown of IGF2BP1 promoted HSP27 phosphorylation at Ser-78 and Ser-82, while overexpressing IGF2BP1 decreased ERK4 and phosphoHSP27 levels. While phosphoThr-182 of MK5, which is required for MK5 activation, was not examined, the authors showed that siRNA-mediated depletion of MK5, as well as inhibition of MK2/MK3/MK5 by Hsp25 kinase inhibitor reduced HSP27 phosphorylation. Phosphorylation of HSP27 controls actin dynamics [reviewed in [75], and indeed IGF2BP1 knockdown-triggered changes in the actin cytoskeleton were abolished by both depletion and inhibition of MK5 [69]. The authors used time-lapse microscopy to demonstrate that IGF2BP1 promoted the velocity of cell migration by inhibiting MK5-mediated phosphorylation of HSP27. MK5-induced HSP27 phosphorylation leads to a significant decrease in cell migration velocity. Thus, overexpression of IGF2BP1 protein in cancer cells results in inhibition of ERK4 mRNA translation and activation of MK5, and subsequent reduction in HSP27 phosphorylation. This promotes the velocity of tumour cell migration. Interestingly, tumour cells often display elevated HSP27 levels [reviewed in [75]. Thus increased IGF2BP1 and HSP27 protein levels both promote cell migration/invasiveness.

MK5 may exert anti- and pro-oncogenic effects as demonstrated by two studies from the group of Sun. In a first study, this group described that MK5 knockout mice were more susceptible to 7,12-dimethylbenzathracene (DMBA)-induced skin cancer [46]. The authors postulated that MK5 suppresses the initiation stage of skin carcinogenesis by mediating oncogene-induced senescence. The exact mechanism by which MK5 can act as a negative regulator in oncogenic RAS proliferative signalling is incompletely understood, but MK5’s ability to suppress cell proliferation relies on intact kinase activity and nuclear localization, because neither kinase dead mutants nor mutants that reside in the cytoplasm can prevent oncogenic RAS-induced cell proliferation [45,46]. The authors provided evidence that MK5 transactivates p53 through phosphorylation of Ser-37, resulting in increased expression of p21^Cip-1^ and subsequently cell cycle arrest [46]. Additionally, MK5 inhibited RAS-induced JNK activity by 85% [44]. The JNK pathway is implicated in proliferation and can act as a negative regulator of the p53 tumour suppressor [76,77]. These observations suggest a dual pathway by which MK5 prevents oncogenic RAS-induced cell proliferation. The fine molecular mechanism by which MK5 interferes with the JNK pathway is not known, nor has a functional link in cancer been established.

In a later study, the same group showed that once the tumour is established, tumour growth was drastically impaired in MK5^−/−^ mice, while sustained growth and malignant progression of tumours was observed in MK5^+/+^ and MK5^+/−^ mice [78]. MK5 supports tumour growth and progression by stimulating angiogenesis. MK5 mediates endothelial cell migration in response to vascular endothelial growth factor (VEGF) and maybe additional factors secreted by the skin tumour epithelium (Figure 3). VEGF binds predominantly VEGF receptor 2 (VEGFR2) and this triggers MK5 Thr-182 phosphorylation through a cascade involving the p38^MAPK^ isoforms α and β, but not ERK3 and ERK4 [78]. Activation of the p38^MAPK^ pathway by VEGF has been shown to induce phosphorylation of HSP27 and to provoke actin reorganization and migration of endothelial cells [79]. Although HSP27 is a genuine substrate of MK5 [80] and MK5 can be activated by p38^MAPK^, VEGF-induced HSP27 phosphorylation was not affected in MK5 depleted human vascular umbilical veil endothelial cells. Focal adhesion kinase (FAK) is another major regulator of cytoskeletal organization and cell migration [81]. Yoshizuka and colleagues demonstrated that VEGFR2-induced MK5 activation is essential for FAK activation and cytoskeletal reorganization during migration of endothelial cells [78]. The exact mechanism for MK5-mediated FAK phosphorylation and activation remains to be solved.

A siRNA screen against the complete human kinome revealed an increase in c-MYC protein levels in cells treated with MK5 siRNA [82]. The authors went on to show that MK5 induced expression of the microRNAs miR-34b and miR-34c by phosphorylating the transcription factor FOXO3a which binds the pre-miR-34 promoter. MK5 predominantly phosphorylates FOXO3a at Ser-215 *in vivo* (and at other sites *in vitro* as well) and MK5-mediated phosphorylation of this site is required for the upregulation of miR-34b/c levels. The miR-34b/c binds c-*myc* mRNA, resulting in reduced c-MYC protein levels. MYC protein seems to be engaged in a negative feedback loop by binding to the MK5 promoter and enhancing expression of MK5 [82]. Because MYC is a central regulator of the cell cycle and aberrant MYC expression plays a central role in oncogenesis [83], Kress and colleagues investigated the state of MK5 expression in tumour cells with increased MYC levels. MK5 expression was higher in normal colon mucosa than in colorectal carcinoma, whereas MYC expression was weak in normal colon epithelium, but strong in colorectal tumour tissues. Moreover, low levels of MK5 mRNA were associated with increased probability of the development of distant metastasis [82]. These exciting findings point to a tumour-suppressive function of MK5. It would be interesting to validate the MK5-MYC link in *mk5*^−/−^ mice or in transgenic mice overexpressing an active MK5 mutant by examining Myc levels and the development of colon cancer in these animals compared to wild-type mice. FOXO3a was recently shown to repress the *VEGF* promoter and reduce expression of VEGF [84]. FOXO3a also stimulates expression of miR34b, a microRNA that causes cell cycle G1 arrest and suppresses cell invasion of melanoma cells [85,86]. Hence, MK5 may through FOXO3a-mediated phosphorylation promote angiogenesis by enhancing the expression of VEGF, but reduce invasiveness by upregulation of miR-34b.

## Conclusions

*In vitro* studies and animal models demonstrate that the ERK3/4-MK5 pathway can participate in several processes that are dysregulated in cancer, including cell proliferation, cell motility, invasiveness, and angiogenesis. However, a bona fide role for ERK3, ERK4 and MK5 in human cancer remains to be elucidated. ERK3, ERK4 and MK5 seem to act downstream of the Ras/Raf pathway or to be implicated in the regulation of c-Myc expression. The *rasraf* and c-*myc* genes are amongst the most commonly mutated genes in cancer [58,87,88]. The lack of potent Ras and c-Myc inhibitors and the acquirement of drug resistance make ERK3, ERK4, and MK5 attractive targets for therapeutic drugs in tumours with perturbed RAS, RAF or c-MYC activity. ERK3 was upregulated in doxorubicin-resistent breast cancer MCF-7 cells and can be a potential target for anti-cancer drug development [89]. Because MK5 can operate as a tumour suppressor and a tumour promoter, drugs that hamper or augment MK5’s activity should be designed. Some selective MK5 inhibitors have been identified, but they have not been tested in clinical trials [90-92]. No MK5 activators to stimulate its tumour suppressor property are available, nor have any compounds that modulate the activity of ERK3 and ERK4 been developed.

## Abbreviations

DMBA, 7,12-dimethylbenzathracene; ERK, Extracellular signal-regulated kinase; IGF2BP1, Insulin-like growth factor 2 mRNA-binding protein 1; MAPK, Mitogen-activated protein kinase; MAPKAPK, MAPK-activated protein kinases; PRAK, p38 regulated/activated protein kinase; VEGF, Vascular endothelial growth factor; VEGFR2, VEGF receptor 2.

## Competing interests

The authors declare that they have no competing interests.

## Authors’ contributions

SK, GD, and UM contributed in drafting the manuscript and revising its scientific content. All authors read and approved the final manuscript.
